# Liver X Receptor Agonist Therapy Prevents Diffuse Alveolar Hemorrhage in Murine Lupus by Repolarizing Macrophages

**DOI:** 10.3389/fimmu.2018.00135

**Published:** 2018-02-02

**Authors:** Shuhong Han, Haoyang Zhuang, Stepan Shumyak, Jingfan Wu, Chao Xie, Hui Li, Li-Jun Yang, Westley H. Reeves

**Affiliations:** ^1^Division of Rheumatology & Clinical Immunology, Department of Medicine, University of Florida, Gainesville, FL, United States; ^2^Department of Pathology, Immunology, and Laboratory Medicine, University of Florida, Gainesville, FL, United States

**Keywords:** lupus, diffuse alveolar hemorrhage, therapy, inflammation, macrophage polarization, liver X receptors, hypoxia-inducible factor 1-α

## Abstract

The generation of CD138^+^ phagocytic macrophages with an alternative (M2) phenotype that clear apoptotic cells from tissues is defective in lupus. Liver X receptor-alpha (LXRα) is an oxysterol-regulated transcription factor that promotes reverse cholesterol transport and alternative (M2) macrophage activation. Conversely, hypoxia-inducible factor 1-α (HIF1α) promotes classical (M1) macrophage activation. The objective of this study was to see if lupus can be treated by enhancing the generation of M2-like macrophages using LXR agonists. Peritoneal macrophages from pristane-treated mice had an M1 phenotype, high HIFα-regulated phosphofructokinase and TNFα expression (quantitative PCR, flow cytometry), and low expression of the LXRα-regulated gene ATP binding cassette subfamily A member 1 (*Abca1*) and *Il10* vs. mice treated with mineral oil, a control inflammatory oil that does not cause lupus. Glycolytic metabolism (extracellular flux assays) and *Hif1a* expression were higher in pristane-treated mice (M1-like) whereas oxidative metabolism and LXRα expression were higher in mineral oil-treated mice (M2-like). Similarly, lupus patients’ monocytes exhibited low LXRα/ABCA1 and high HIF1α vs. controls. The LXR agonist T0901317 inhibited type I interferon and increased ABCA1 in lupus patients’ monocytes and in murine peritoneal macrophages. *In vivo*, T0901317 induced M2-like macrophage polarization and protected mice from diffuse alveolar hemorrhage (DAH), an often fatal complication of lupus. We conclude that end-organ damage (DAH) in murine lupus can be prevented using an LXR agonist to correct a macrophage differentiation abnormality characteristic of lupus. LXR agonists also decrease inflammatory cytokine production by human lupus monocytes, suggesting that these agents may be have a role in the pharmacotherapy of lupus.

## Introduction

Mice with pristane-induced lupus develop an autoimmune syndrome closely resembling systemic lupus erythematosus (SLE) with lupus-specific autoantibodies, nephritis, arthritis, diffuse alveolar hemorrhage (DAH), and hematological manifestations (1). Pristane-induced lupus in C57BL/6 (B6) mice is the only model of lupus-associated DAH (2, 3), an often fatal complication seen in ~3% of SLE patients (4). DAH in pristane-induced lupus is associated with antineutrophil cytoplasmic antibody negative pulmonary capillaritis and is mediated by macrophages (Mϕ) (3).

Pristane-treated mice develop lupus in the setting of non-resolving inflammation (5), which may result in part from impaired clearance of dead cells (6). CD11b^+^F4/80^+^Ly6C^hi^ inflammatory Mϕ (Ly6C^hi^ Mϕ) accumulate in the peritoneum after pristane injection (6, 7). In contrast, peritoneal exudate cells (PEC) from mice treated with mineral oil (MO), an inflammatory hydrocarbon that does not cause lupus, are progressively enriched in a subset of anti-inflammatory CD11b^+^F4/80^+^CD138^+^ Mϕ reminiscent of alternatively activated (M2) Mϕ (6). CD138^+^ Mϕ are highly phagocytic for apoptotic cells and their deficiency in pristane-treated mice may promote non-resolving inflammation resulting in end-organ damage.

Although an over-simplification (8, 9), bone marrow (BM)-derived Mϕ are classified as classically activated (M1) or alternatively activated (M2). Murine M1 Mϕ express high levels of Ly6C, CD80/CD86, CD274 (PD-L1), and CCR2 and produce TNFα, IL-1β, and IL-12. In contrast, M2 Mϕ express Fizz1 (*Retnlb*), Ym1 (*Chil3*), Arginase 1 (*Arg1*), CD206 (*Mrc1*), CD273 (PD-L2, *Pdcd1lg2*), scavenger receptors, CX_3_CR1, and low levels of Ly6C and produce TGFβ and IL-10 (10). Phosphorylation of the transcription factor CREB promotes M2 Mϕ polarization (11). CD138^+^ Mϕ from MO-treated mice express M2 activation markers and have high levels of p-CREB (6). The present study addresses the role of two additional transcription factors, liver X receptor-alpha (LXRα) and hypoxia inducible factor 1-alpha (HIF1α), in lupus.

Liver X receptor-alpha, an oxysterol-regulated transcription factor activated via the endosome/lysosome associated Lamtor1-mTORC1 pathway, helps determine whether or not M0 Mϕ polarize to M2 (12, 13). Oxysterols derived from the phagocytosis of apoptotic cells activate the LXR pathway in Mϕ, upregulating genes involved in the recognition of dead cells (*Mertk*) and cholesterol efflux (e.g., ATP binding cassette A1, *Abca1*) and downregulating proinflammatory gene expression (14). Along with their dependence on LXRα, M2 Mϕ rely on oxidative phosphorylation and fatty acid oxidation to fuel mitochondrial oxidative metabolism whereas M1 Mϕ rely on glycolysis (15, 16). M1 polarization is promoted by HIF1α, a key regulator of glycolytic metabolism (15, 17, 18), which upregulates glycolytic enzymes, proinflammatory cytokines, and expression of the M1 marker CD274 (17). We show that an imbalance between LXRα and HIF1α activity is involved in the pathogenesis of end-organ damage (DAH) in lupus. Therapy with an LXR agonist corrected this imbalance and prevented DAH.

## Materials and Methods

### Mice

B6 mice (Jackson) maintained under specific pathogen free conditions were injected with pristane (Sigma-Aldrich, 0.5 ml i.p.), mineral oil (MO; C.B. Fleet Co.), PBS, or left untreated. PEC were collected 14 days later. Some mice were treated with pristane on d0 plus either LXR agonist T0901317 (200 µg in DMSO per mouse i.p. daily) or DMSO alone. Mice received T0901317 on d1–d14 or on d1–d3, d3–d14, or d7–d14 only. On d14, lungs were evaluated for DAH by gross inspection of the excised lungs followed by microscopic confirmation as described previously (3). This study was carried out in accordance with the recommendations of the Animal Welfare Act and US Government Principles for the Utilization and Care of Vertebrate Animals and was approved by the UF IACUC.

### Patients and Healthy Donors

For flow cytometry and isolation of peripheral blood mononuclear cells (PBMCs), heparinized blood was obtained from 22 SLE patients meeting ACR criteria who were seen consecutively in the UF Autoimmune Disease Clinic (19) and 24 matched healthy donors with no autoimmune disease. For RNA isolation, blood was collected in PAXgene tubes (BD Biosciences). SLE activity was assessed using the SLEDAI (20). This study was carried out in accordance with the recommendations of the International Committee of Medical Journal Editors and was approved by the UF IRB. All subjects gave written informed consent in accordance with the Declaration of Helsinki.

### Quantitative PCR

Quantitative PCR (Q-PCR) was performed as described (21) using RNA extracted from 10^6^ mouse PEC (TRIzol, Invitrogen). RNA was isolated from human blood with the QIAamp RNA Blood Mini Kit (Qiagen). cDNA was synthesized using the Superscript II First-Strand Synthesis kit (Invitrogen). SYBR Green Q-PCR analysis was performed using an Opticon II thermocycler (Bio-Rad). Gene expression was normalized to 18 S RNA, and the expression level was calculated using the 2^-Δ ΔCt^ method. Primer sequences are in Table 1.

**Table 1:** Primer sequences.

Gene	Forward primer (5′ → 3′)	Reverse primer (5′ → 3′)
18 S	AGGCTACCACATCCAAGGAA	GCTGGAATTACCGCGGCT
**Human**		
*NR1H3* (LXRα)	ACTCGAAGATGGGGTTGATG	GGAGGTACAACCCTGGGAGT
*ABCA1*	AACAAGCCATGTTCCCTCAG	GACGCAAACACAAAAGTGGA
*MX1*	CACGAGAGGCAGCGGGATCG	CCTTGCCTCTCCACTTATCTTC
*LY6E*	AGGCTGCTTTGGTTTGTGAC	AGCAGGAGAAGCACATCAGC
*HIF1A*	TCCATGTGACCATGAGGAAA	TCTTCCTCGGCTAGTTAGGG
*PFKL*	CTCCTCGCCCACCAGAAG	CTGTGTGTCCATGGGAGATG
*HK2*	TCTATGCCATCCCTGAGGAC	AAACCCAGTGGGAGCTTCTT
**Mouse**		
*Nr1h3*	TGGAGAACTCAAAGATGGGG	TGAGAGCATCACCTTCCTCA
*Abca1*	GCTGCAGGAATCCAGAGAAT	CATGCACAAGGTCCTGAGAA
*Hif1a*	TCCATGTGACCATGAGGAAA	GGCTTGTTAGGGTGCACTTC
*Mx1*	GATCCGACTTCACTTCCAGATGG	CATCTCAGTGGTAGTCCAACCC
*Il10*	GGTTGCCAAGCCTTATCGGA	ACCTGCTCCACTGCCTTGCT
*Tnfa*	CATCTTCTCAAAATTCGAGTGACAA	TGGGAGTAGACAAGGTACAACCC
*Chil3*	TGTACCAGCTGGGAAGAAAC	GAGAGCAAGAAACAAGCATGG
*G6pd*	CCCCCACAGTCTATGAAGCA	TGGTTCGACAGTTGATTGGA
*Pfkl*	GGGCTGATTGGCTATTCATT	TGATGATGTTCAGCCGAGAG
*Hk2*	GGGTTTCACCTTCTCCTTCC	TTCAGCAAGGTGACCACATC

### Culture of Adherent Peripheral PBMC-Derived Monocytes

Peripheral blood mononuclear cells from lupus patients and healthy donors were isolated from heparinized blood by density gradient centrifugation (Ficoll-Hypaque, GE Healthcare Bio-Sciences). PBMCs were incubated at 37°C for 1 h in AIM-V medium (Invitrogen), and non-adherent cells were removed. Adherent cells (90–95% CD14^+^) were lysed with RLT lysis buffer (Qiagen) for RNA isolation. Monocytes were cultured with LXRα agonist GW3965 (1 µM, Sigma-Aldrich), for 24 h in AIM-V medium before isolating RNA. Gene expression was measured by Q-PCR. In some experiments, monocytes were treated with IFNα (1,000 U/ml) (R&D Systems) for 1 h, followed by addition of LXR agonists (GW3965 or T0901317, 1 µM in DMSO), or DMSO alone, and then cultured for 24 h. Some cells were lysed for RNA isolation. The remaining cells were analyzed by flow cytometry. About 10–50,000 events per sample were acquired using an LSRII flow cytometer (BD-Biosciences) and analyzed with Flowjo software (Tree Star Inc.).

### Flow Cytometry and Sorting of Mouse Mϕ

Flow cytometry was performed as described (21) using anti-mouse CD16/32 (Fc Block; BD Biosciences) before staining with primary antibody or isotype controls. Cells were surface-stained, then fixed/permeabilized (Fix-Perm buffer, eBioscience) before intracellular staining. Antibodies are listed in Table 2. Uptake of low-density lipoproteins was assessed by incubating PEC with BODIPY-labeled LDL (10 µg/ml, Invitrogen) (16). Data were acquired and analyzed as above. CD11b^+^Ly6C^hi^ LyG^-^ and CD11b^+^CD138^+^ Ly6G^-^cells were sorted using a FACSaria cell sorter and 40,000 cells/subset were lysed immediately for RNA extraction.

**Table 2:** Antibodies used for flow cytometry.

Specificity (clone)	Fluorochrome	Source
Mouse CD273 (TY25)	Phycoerythrin	Biolegend
Mouse CDE274 (10F.9G2)	Phycoerythrin	Biolegend
Mouse CD138 (281-2)	Phycoerythrin; Allophycocyanin	Biolegend
Mouse CD11b (M1/70)	Brilliant violet-421	Biolegend
Mouse Ly6C (HK1.4)	Allophycocyanin-Cy7	Biolegend
Mouse Ly6G (1A8)	Phycoerythrin	BD Bioscience
Mouse CD80 (16-10A1)	PerCP-Cy5.5	Biolegend
Mouse CD86 (GL-1)	Allophycocyanin-Cy7	Biolegend
Mouse CD36 (HM36)	Phycoerythrin	Biolegend
Mouse TNFα (MP6-XT22)*	Allophycocyanin	Biolegend
Mouse/human ABCA1 (5A1-1422.22)^a^	Allophycocyanin	Novus Biologicals
Human CD14 (MϕP9)	PerCP	BD Bioscience
Human CD16 (3G8)	Fluorescein isothiocyanate	BD Bioscience
Human CD64 (10.1)	Phycoerythrin	eBioscience
Human PFKL (polyclonal)	Fluorescein isothiocyanate	Aviva Systems Biology

*^a^Intracellular staining*.

### Extracellular Flux Analysis

For real-time analysis of mitochondrial oxygen consumption rate (OCR) and extracellular aerobic acidification rate (ECAR), peritoneal adherent cells and FACS-sorted Ly6C^hi^ Mϕ and CD138^+^ Mϕ were analyzed with an XF-96 Extracellular Flux Analyzer (Seahorse Bioscience) (16). Briefly, peritoneal cells were collected by lavage from mice treated with pristane or MO for 14 days and stained with antibodies against CD11b, Ly6G, Ly6C, and CD138 (Table 2). CD11b^+^Ly6G^-^Ly6C^hi^ Mϕ and CD11b^+^Ly6G^-^CD138^+^ Mϕ were sorted using a FACSAira II Cell Sorter (BD Biosciences). A total of 5 × 10^4^ peritoneal cells, Ly6C^hi^ Mϕ, or CD138^+^ Mϕ were resuspended in AIM-V medium (Thermo Fisher) and placed into 96-well XF cell culture microplates (Seahorse Bioscience). Two hours later, the cells were washed three times with warm XF assay medium and cultured in XF assay medium. Three or more consecutive measurements were obtained under basal conditions and after sequential addition of 1 µM oligomycin, 0.75 µM FCCP (fluoro-carbonyl cyanide phenylhydrazone), and 250 nM rotenone plus 250 nM antimycin A (Sigma-Aldrich).

### Statistical Analysis

Statistical analyses were performed using Prism 6.0 (GraphPad Software). Differences between groups were analyzed by two-sided unpaired Student’s *t*-test unless otherwise indicated in the figure legend. Before comparing the means, we tested for equality of variance using the F-test. If the variances did not differ, we used Student’s *t*-test. If there was statistically significant evidence that the variances differed, we used Welch’s *t*-test. Data were expressed as mean ± SD. Correlation was analyzed using the Pearson correlation coefficient. *p* < 0.05 was considered significant. All experiments in mice were repeated at least twice.

## Results

Diffuse alveolar hemorrhage in pristane-induced lupus is prevented by peritoneal Mϕ (but not neutrophil) depletion (3). In contrast, MO-treated mice do not develop DAH despite their high numbers of peritoneal Mϕ. We have shown recently that pristane treatment favors classical (M1) Mϕ activation whereas MO favors the generation of pro-resolving alternatively activated (M2) Mϕ (6). We examined transcriptional activation in peritoneal Mϕ from pristane- vs. MO-treated mice.

### Pristane Treatment Increases Hif1a

**Figure 1:**
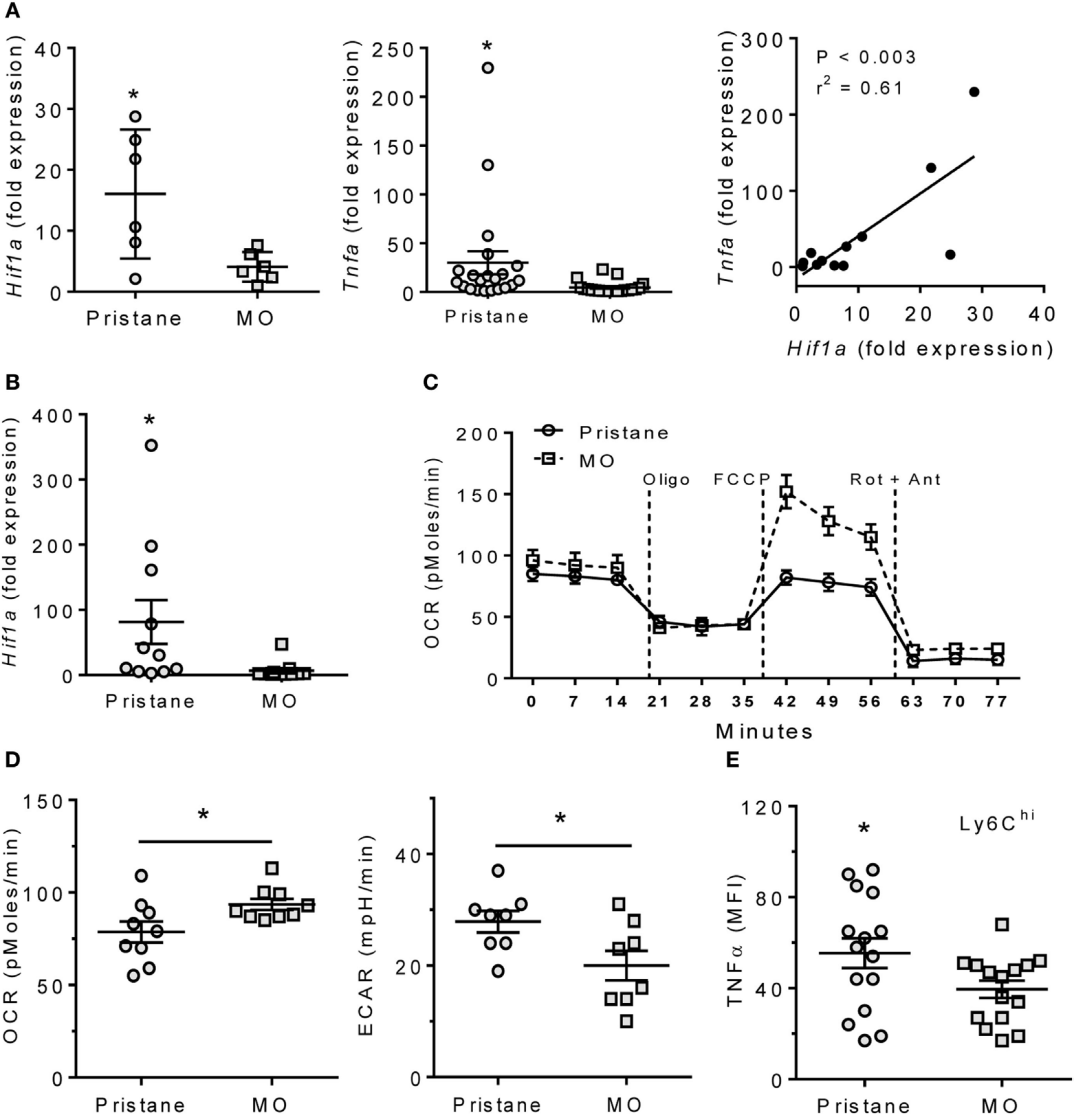
Pristane increases HIF1α, TNFα, and glycolysis. B6 mice were injected i.p. with pristane and MO. Peritoneal cells were collected at d14 and RNA was extracted. **(A)** Expression of *Hif1a* and *Tnfa* mRNA relative to 18 S (Q-PCR). *P < 0.05 vs. control (unpaired Welch’s *t*-test). **(B)** Peritoneal CD11b^+^Ly6C^high^ cells were flow-sorted from pristane- and MO-treated mice, and *Hif1a* expression was measured by Q-PCR. *P = 0.05 vs. control (unpaired Welch’s *t*-test). **(C)** Extracellular flux analysis of adherent peritoneal cells from pristane- and MO-treated mice (14 days after treatment). After 1 h incubation, oxygen consumption rate (OCR) was determined with sequential addition of 1 µg/ml oligomycin (Oligo), 400 nM FCCP, and 1 µM rotenone + 1 µM antimycin A (Rot + Ant). **(D)** Effects of pristane and MO on basal OCR (left) and extracellular acidification rate (ECAR, right) (XF96 Analyzer). Experimental treatments were performed with five technical replicates and three biological replicates. *P < 0.05 vs. control (unpaired Student’s *t*-test). **(E)** Intracellular TNFα staining of CD11b^+^Ly6C^high^ cells from pristane vs. MO treated mice. *P < 0.05 vs. control (unpaired Student’s *t*-test).

M1 Mϕ are highly dependent on glycolytic metabolism, which is regulated by HIF1α (15, 17, 22). In B6 mice, expression of both *Hif1a* and the proinflammatory cytokine *Tnfa* was higher in PEC from pristane- vs. MO-treated mice (Figure 1A). Expression of *Hif1a* and *Tnfa* correlated. As PEC from pristane- (but not MO-) treated mice contain many Ly6C^hi^CD11b^+^F4/80^+^ cells (7), we determined *Hif1a* expression in flow-sorted Ly6C^hi^CD11b^+^ PEC from pristane- and MO-treated mice. Ly6C^hi^ Mϕ from pristane-treated mice exhibited higher levels of *Hif1a* than Ly6C^hi^ Mϕ from MO-treated mice (Figure 1B), suggesting that glycolysis might be more active in Mϕ from pristane- vs. MO-treated mice. The increased ECAR and decreased OCR of PEC from pristane- vs. MO-treated mice in extracellular flux assays supported that hypothesis (Figures 1C,D). Consistent with the correlation between *Tnfa* and *Hif1a* in PEC (Figure 1A), higher *Hif1a* expression in the Ly6C^hi^ Mϕ subset from pristane-treated mice also was associated with higher intracellular staining for TNFα (Figures 1B,E).

### MO Treatment Increases LXR Activity

**Figure 2:**
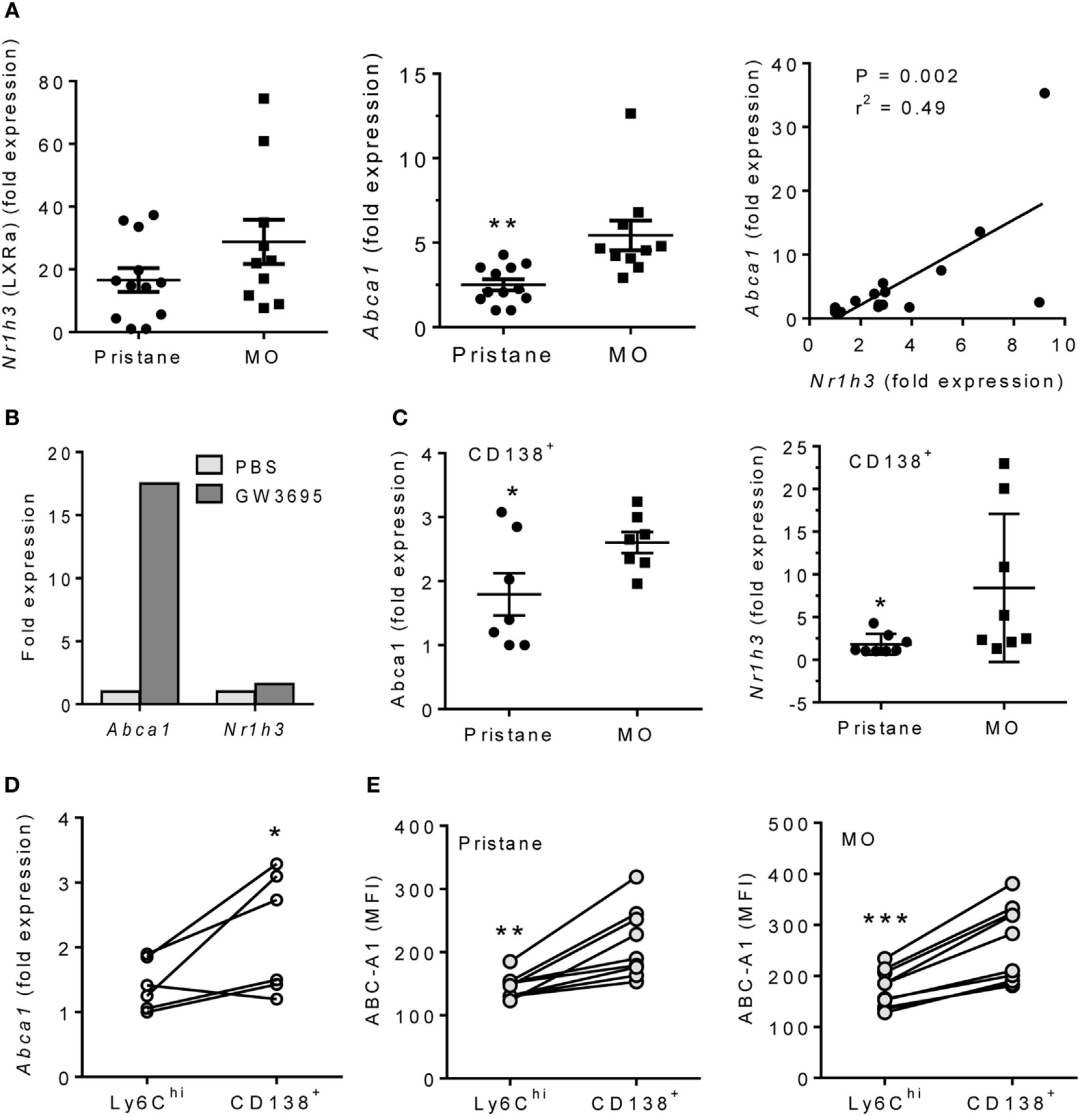
Pristane decreases LXRα activity in PEC. B6 mice were injected i.p. with pristane or MO. PEC were collected at d14 and RNA was isolated. **(A)** Q-PCR for *Nr1h3* and *Abca1* expression relative to 18 S. **P < 0.01, Welch’s *t*-test. **(B)** PEC from wild-type mice were stimulated with 1 µM GW3965 for 24 h. *Nr1h3* and *Abca1* expression levels were determined by Q-PCR (representative of three experiments). **(C)** CD138^+^CD11b^+^ cells from pristane- and MO-treated mice were flow sorted, and mRNA was analyzed by Q-PCR. Left, *Abca1*; right, *Nr1h3*. **P < 0.05, Student’s *t*-test (left) and Welch’s *t*-test (right). **(D)** Peritoneal CD11b^+^Ly6C^hi^ and CD11b^+^CD138^+^ cells from MO-treated mice were flow sorted, and *Abca1* expression was analyzed (Q-PCR). *P < 0.05 (paired Student’s *t*-test). **(E)** Peritoneal cells from pristane- and MO-treated mice were stained with antibodies against CD11b, CD138, Ly6C, and Abca1. Mean Fluorescence Intensity (MFI) of Abca1 staining (flow cytometry) was compared between CD11b^+^Ly6C^hi^ and CD11b^+^CD138^+^ subsets. **P < 0.01; ***P < 0.001 vs. control (paired Student’s *t*-test).

Peritoneal exudate cells from MO-treated mice are enriched in M2 Mϕ (6). As alternatively activated Mϕ which depend on mitochondrial oxidative metabolism (15), the increased OCR and decreased ECAR of MO- vs. pristane-treated Mϕ in extracellular flux assays (Figures 1C,D) suggested an M2-like phenotype. We therefore examined the activity of LXRα, a transcription factor that regulates M2 polarization (13). Expression of *Nr1h3* (encoding LXRα), increased slightly in PEC from MO-treated vs. pristane-treated mice, but it was not statistically significant. However, expression of the LXRα-regulated gene *Abca1* was substantially higher in PEC from MO-treated mice (Figure 2A). Expression levels of *Abca1* and *Nr1h3* correlated. Treatment of PEC from wild-type mice with the LXR agonist GW3695 induced *Abca1* but had only a modest effect on *Nr1h3* expression (Figure 2B). Anti-inflammatory CD138^+^ Mϕ expand in PEC from MO- vs. pristane-treated mice (6). Sorted CD11b^+^CD138^+^ Mϕ from MO-treated mice expressed higher levels of *Abca1* than those from pristane-treated mice and modestly higher levels of *Nr1h3* (Figure 2C). *Abca1* expression was higher in sorted CD138^+^ Mϕ than in Ly6C^hi^ Mϕ from the same mouse (Figure 2D). Intracellular Abca1 protein also was higher in CD138^+^ vs. Ly6C^hi^ Mϕ from both pristane- and MO-treated mice (Figure 2E).

### Phenotypes of CD138^+^ Mϕ from Pristane vs. MO Treated Mice

**Figure 3:**
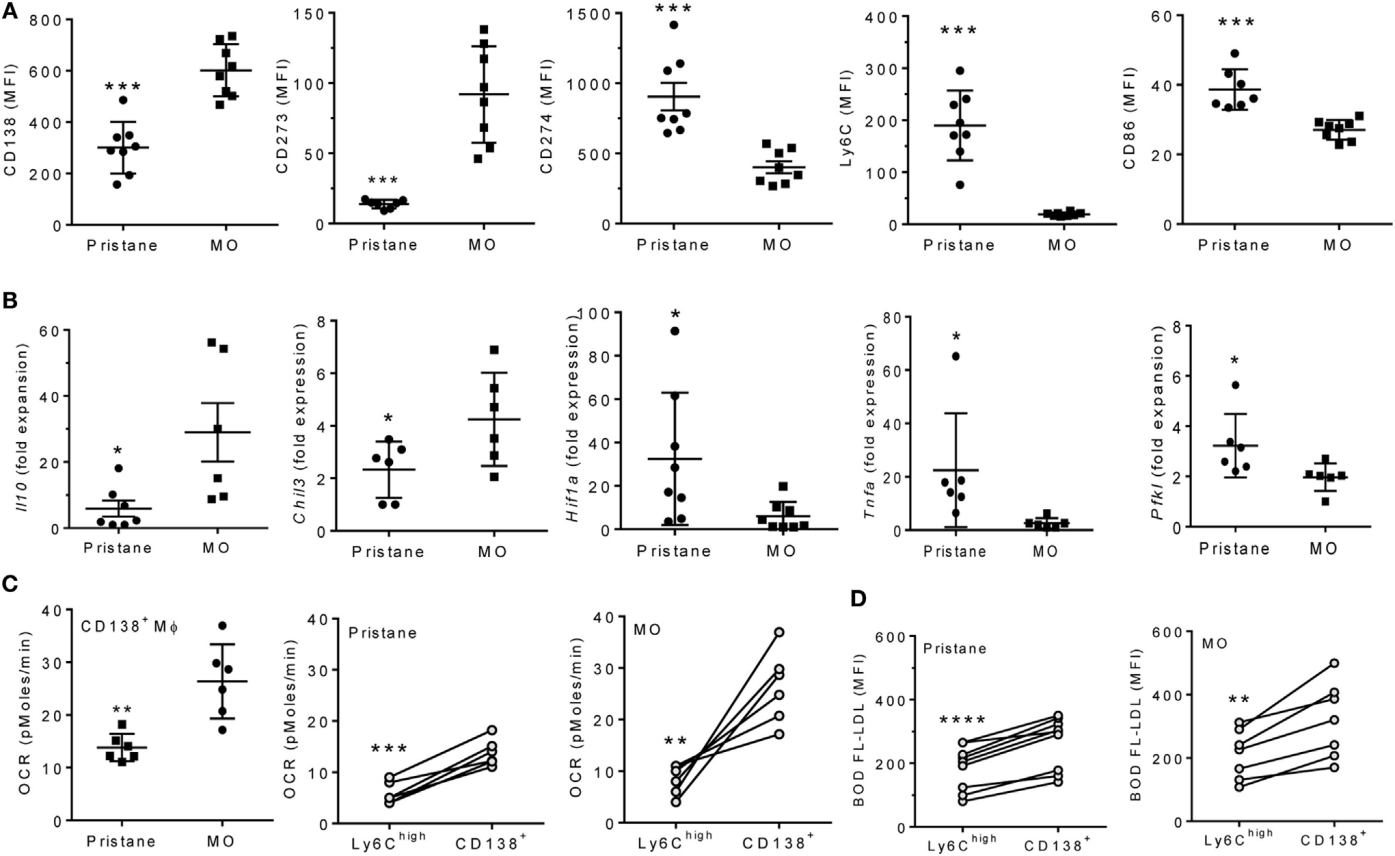
CD138^+^ Mϕ in pristane-treated mice are M1-like. B6 mice were injected i.p. with pristane or mineral oil (MO). Peritoneal exudate cells were collected at d14. **(A)** PEC were stained with antibodies against CD11b, CD138, CD273, CD274, Ly6C, and CD86. CD11b^+^CD138^+^ cells were gated to analyze staining of the other markers.***P < 0.001 by unpaired Student’s *t*-test (panels 1 and 5) or Welch’s *t*-test (panels 2, 3, and 4). **(B)** CD11b^+^CD138^+^ cells were flow sorted, and expression levels of *Il10, Chil3, Tnfa, Hif1a*, and *Pfkl* were determined relative to 18 S (Q-PCR). *P < 0.05 by Student’s *t*-test (panels 2 and 5) or Welch’s *t*-test (panels 1, 3, and 4). **(C)** Peritoneal CD11b^+^Ly6C^hi^ and CD11b^+^CD138^+^ cells were flow sorted from pristane- and MO-treated mice. OCR was measured (XF96 Analyzer). Left, CD138^+^ Mϕ from pristane- vs. MO-treated mice; middle and right, Ly6C^hi^ vs. CD138^+^ Mϕ from individual pristane- and MO-treated mice. Experimental treatments were performed with six technical replicates. **P < 0.01, ***P < 0.001 by Welch’s unpaired *t*-test (left) or Student’s paired *t*-test (middle and right). **(D)** BODIPY-labeled LDL (10 µg/ml) was added to PEC from pristane- and MO-treated mice for 2 h and cells were then stained with anti-CD11b, Ly6C, and CD138. Mean fluorescence intensity (MFI) of BODIPY-LDL was analyzed. Comparison of Ly6C^hi^ vs. CD138^+^ Mϕ from individual pristane- (left) and MO- (right) treated mice **P < 0.01; ****P < 0.0001 vs. control, paired Student’s *t*-test.

Although MO-treatment favors the development of CD138^+^ (pro-resolving) rather than Ly6C^hi^ Mϕ (6, 7), surface staining unexpectedly revealed that the phenotypes of CD138^+^ Mϕ from pristane- and MO-treated mice were not identical (Figure 3A). CD138 staining and staining for the M2 Mϕ marker CD273 were higher in MO- than pristane-treated mice. Conversely, staining for the M1 marker CD274, Ly6C, and CD86 was higher in CD138^+^ Mϕ from pristane- vs. MO-treated mice (Figure 3A). By Q-PCR (Figure 3B, CD138^+^ Mϕ from MO-treated mice expressed more *Il10* and *Chil3* (Ym1) and less *Hif1a, Pfkl* (phosphofructokinase, HIF1α-regulated), and *Tnfa* than CD138^+^ Mϕ from pristane-treated mice. In addition, sorted CD138^+^ Mϕ from MO-treated mice exhibited a higher OCR than CD138^+^ Mϕ from pristane-treated mice (Figure 3C, left). In both pristane- and MO-treated mice, the OCR was higher in CD138^+^ Mϕ than in Ly6C^hi^ Mϕ (Figure 3C, middle and right). A similar pattern (higher in CD138^+^ vs. Ly6C^hi^ Mϕ) was seen after staining PEC from pristane vs. MO-treated mice with BODFL-LDL to assess uptake of exogenous LDL (Figure 3D). Overall, CD138^+^ Mϕ from MO-treated mice were more M2-like than the CD138^+^ Mϕ subset from pristane-treated mice and in comparison with the Ly6C^hi^ subset, CD138^+^ Mϕ were more M2-like.

### Inverse Relationship of HIF-1α and LXRα Expression in Lupus Mice

**Figure 4:**
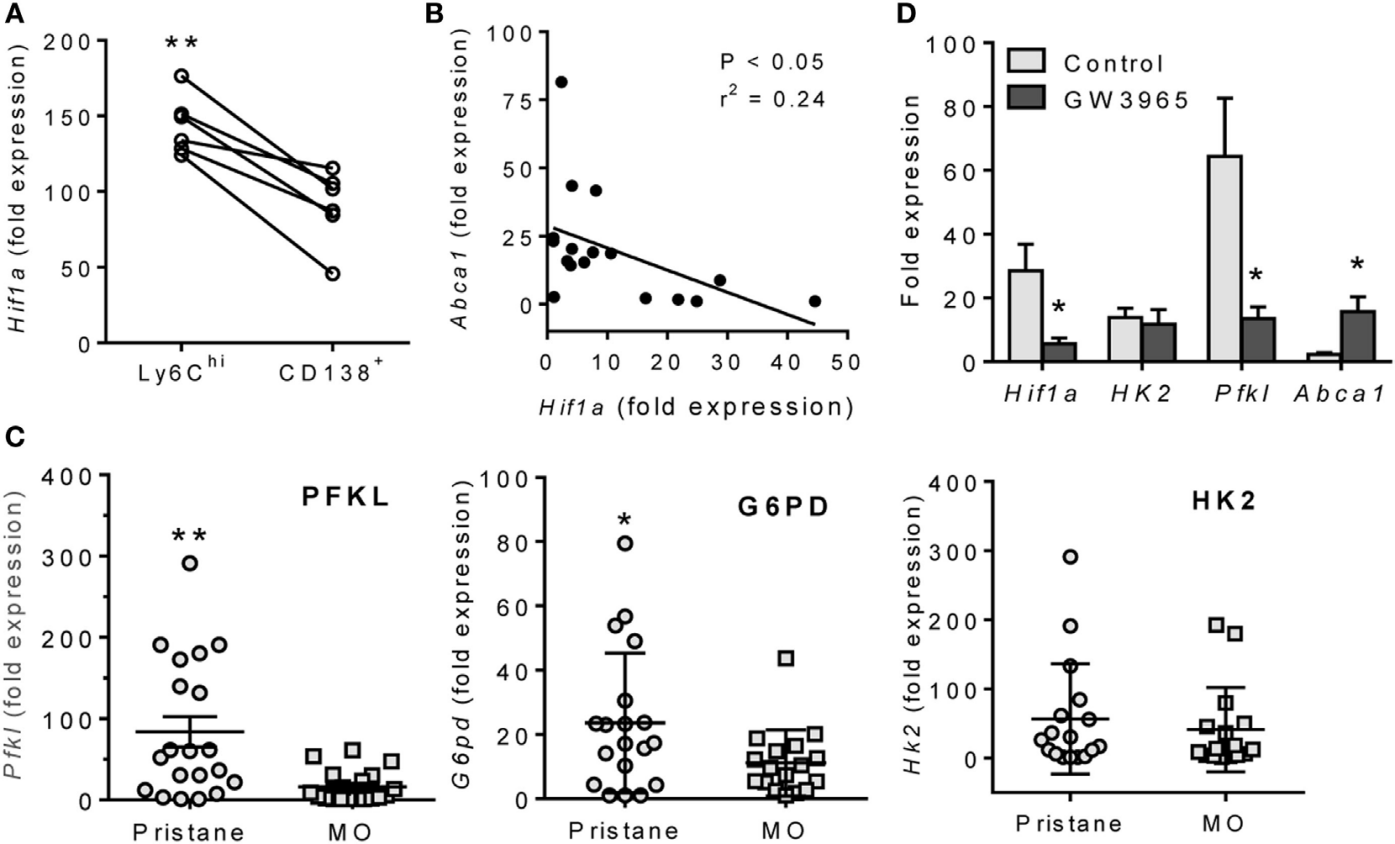
Inverse relationship of LXRα and HIF-1α. **(A)** Peritoneal Ly6C^hi^ and CD138^+^ Mϕ were flow sorted from pristane-treated mice, and *Hif1a* mRNA expression was measured relative to 18 S (Q-PCR). **P < 0.01, Student’s paired *t*-test. **(B)** Inverse relationship of *Hif1a* and *Abca1* mRNA levels in PEC from pristane-treated mice. **(C)** PEC were collected 14 days after pristane- or MO-treatment and expression of HIF1α-regulated genes (*Pfkl, G6pd*, and *Hk2*) was measured (Q-PCR) (*P < 0.05, unpaired Welch’s *t*-test). **(D)** Adherent peritoneal cells from pristane-treated mice were incubated with GW3965 or DMSO for 24 h, and expression levels of *Hif1a, Hk2, Pfkl*, and *Abca1* mRNA were measured relative to 18 S (Q-PCR). (*P < 0.05, **P < 0.01, unpaired Welch’s *t*-test).

Although CD138^+^ Mϕ from lupus (pristane-treated) mice were more “inflammatory” than those from MO-treated controls, *Hif1a* expression was still higher in peritoneal M1-like Ly6C^hi^ than in M2-like CD138^+^ Mϕ from pristane-treated mice (Figure 4A). *Hif1a* mRNA levels correlated inversely with *Abca1* in pristane-treated mice (Figure 4B). Expression of the HIF-1α regulated genes *Pfkl* (23, 24) and *G6pd* (glucose-6-phosphate dehydrogenase) (25) (but not *Hk2*) was higher in pristane- vs. MO-treated mice (Figure 4C). To see if LXR activation downregulates *Hif1a*, peritoneal Mϕ from pristane-treated mice were treated for 24 h with the LXR agonist GW3965, which decreased expression of *Hif1a* as well as *Pfkl*, but not hexokinase-2 (*Hk2*) (Figure 4D). As expected, expression of the LXR-regulated *Abca1* gene increased after GW3965 treatment. These data suggested that treatment with LXR agonists might normalize HIF-1α activity in Mϕ from pristane-treated mice. We therefore examined the possibility of treating DAH using LXR agonists to induce Mϕ repolarization.

### LXR Agonist Therapy Prevents DAH

**Figure 5:**
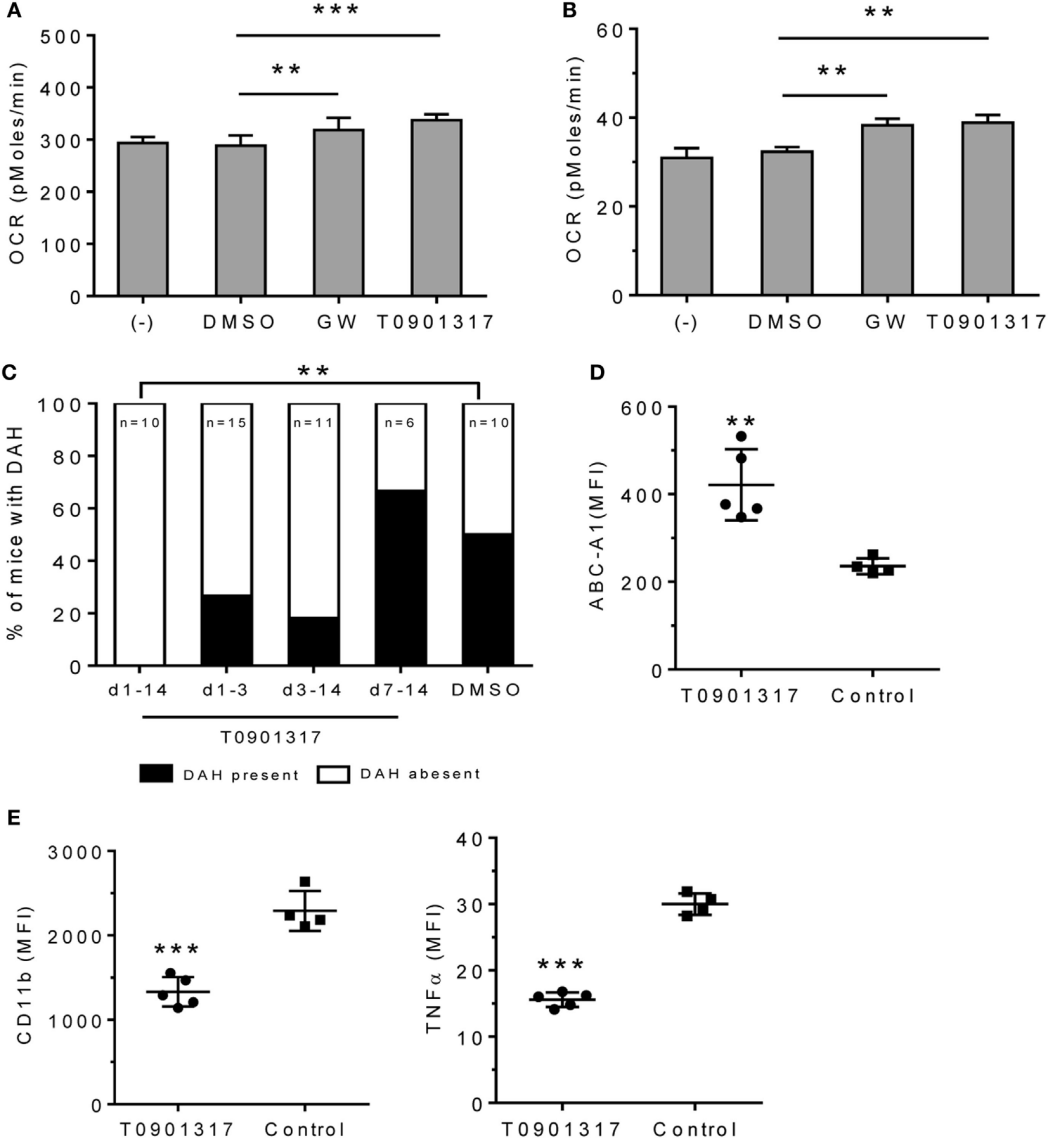
Effect of LXRα agonist on pristane-induced lung hemorrhage. **(A)** *In vitro* treatment of RAW-264.7 cells with GW3965 (GW, 1 µM), T0901317 (1 µM), or DMSO for 24 h. Oxygen consumption rate (OCR) was measured (XF96 Analyzer). Experimental treatments were performed with six technical replicates. **P < 0.01; ***P < 0.001 vs. control (Student’s unpaired *t*-test). **(B)** Adherent peritoneal Mϕ from pristane-treated B6 mice were incubated for 24-h with GW3965, T0901317, or DMSO followed by measurement of OCR. Experimental treatments were performed with six technical replicates. **P < 0.01 vs. control (Student’s unpaired *t*-test). **(C–E)**, B6 mice were injected once with pristane and treated i.p. with T0901317 (200 μg/mouse/day) or DMSO (*n* = 10) starting on the day of pristane treatment. One group received T0901317 daily from d1–d14 (*n* = 10), another from d1–d3 (*n* = 15), another from d3–d14 (*n* = 11), and another from d7–d14 (*n* = 6). **(C)** Frequency of lung hemorrhage in the four groups. 5/10 control mice and 0/10 mice treated with T0901317 (d1–d14) developed DAH (**P < 0.01, χ^2^). **(D, E)** Flow cytometry of CD11b^+^CD138^+^ Mϕ from mice treated with pristane plus T0901317 (d1–d14) vs. DMSO (Control). MFI, mean fluorescence intensity. **(D)** Intracellular staining for Abca1 in CD11b^+^CD138^+^ cells. **P < 0.01 vs. control (Welch’s unpaired *t*-test). **(E)**, Surface staining for CD11b and intracellular staining for TNFα. CD11b^+^CD138^+^ cells were gated to analyze the expression level (MFI) of CD11b and TNFα. *P < 0.05; ***P < 0.001 vs. control (Student’s unpaired *t*-test).

LXR agonists include naturally occurring oxysterols and synthetic ligands, such as GW3965 and T0901317 (26). *In vitro* treatment with GW3965 or T0901317 increased OCR in RAW-264.7 cells (Figure 5A) and adherent peritoneal Mϕ from pristane-treated mice (Figure 5B), suggesting that LXR activation promotes alternative activation.

We treated B6 mice with pristane (d0) plus daily injections of either T0901317 or vehicle and assessed DAH at d14. Daily T0901317 treatment for 14 days completely protected the mice from lung hemorrhage (Figure 5C). Mice treated from d1–d3 or d–d14 may exhibit partial protection, but this did not reach statistical significance. Treatment from d7–d14 had no effect. As expected, intracellular Abca1 staining was higher in CD11b^+^CD138^+^ Mϕ from T0901317-treated mice than in controls (Figure 5D). T0901317 also decreased surface CD11b and intracellular TNFα staining in CD11b^+^CD138^+^ Mϕ (Figure 5E).

### Expression of HIF-1α and LXRα in SLE Patients

**Figure 6:**
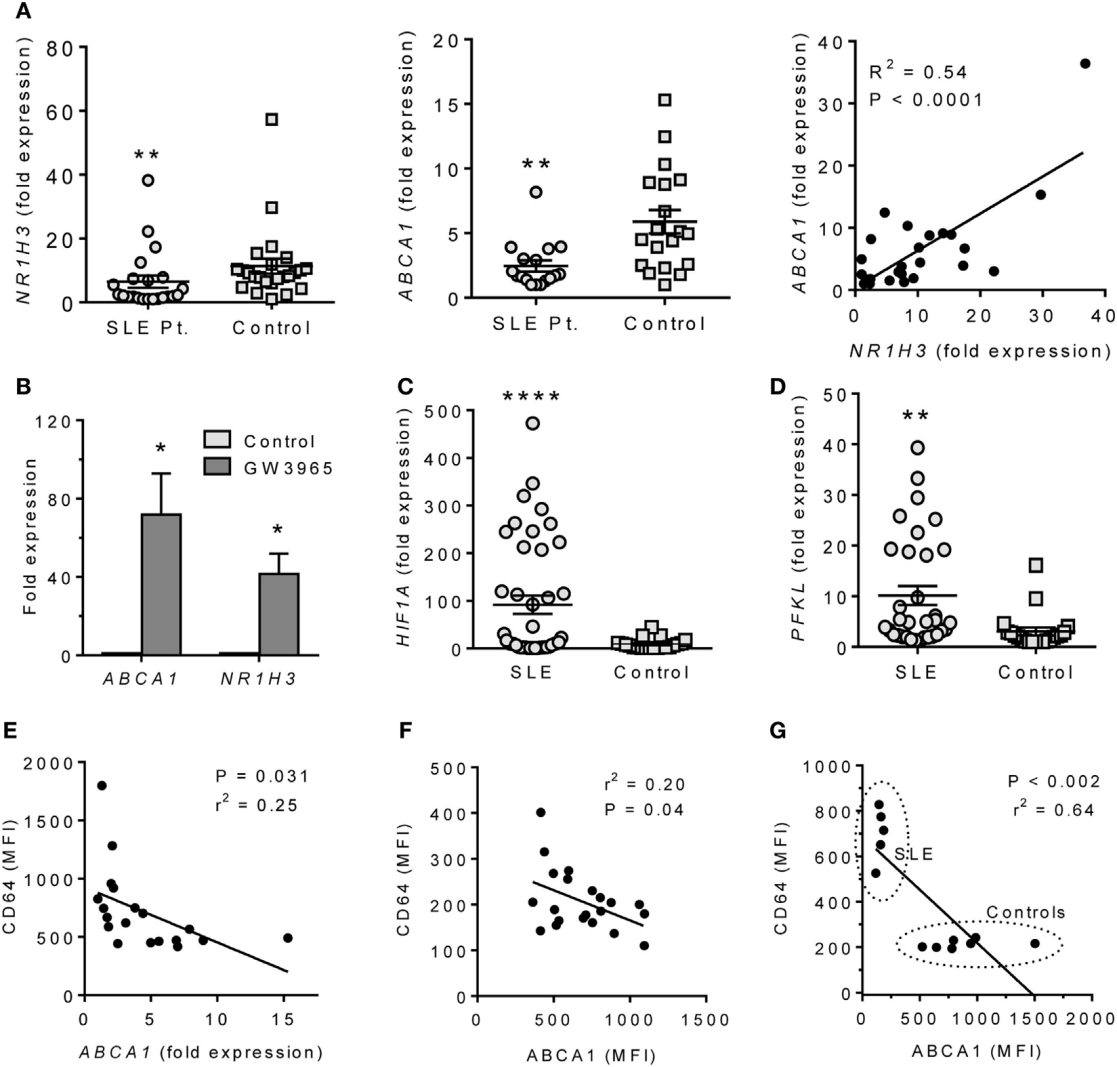
ABCA1 and HIF1α expression in monocytes from SLE patients. **(A)** Expression of *NR1H3* and *ABCA1* in adherent PBMC (Q-PCR) and bivariate analysis of *ABCA1* vs. *NR1H3* (right). Left **P < 0.01 (Student’s unpaired *t*-test); Middle, **P < 0.01 vs. control (Welch’s unpaired *t*-test). **(B)** Adherent PBMCs were treated with 1 µM GW3965 or vehicle alone (Control) for 24 h. *ABCA1* and *NR1H3* expression levels were measured by Q-PCR. *P < 0.05 (Welch’s unpaired *t*-test). **(C)** Expression of *HIF1A* in adherent PBMCs from SLE patients vs. healthy controls (Q-PCR). *P < 0.0001 (Welch’s unpaired *t*-test). **(D)** *PFKL* expression on adherent PBMCs from SLE and healthy controls (Q-PCR). *P < 0.01 (Welch’s unpaired *t*-test). **(E)** Flow cytometry of the IFN-regulated protein CD64 staining (MFI, flow cytometry) vs. *ABCA1* mRNA expression (Q-PCR) in monocytes from unselected SLE patients. **(F)** Flow cytometry of CD64 (surface staining) vs. ABCA1 (intracellular staining) in monocytes from unselected SLE patients. **(G)** CD64 vs. ABCA1 staining in PBMCs from five patients with active SLE and seven healthy controls.

The altered expression of LXRα and HIF-1α in mice with pristane-lupus prompted us to look for similar changes in circulating monocytes from SLE patients. *NR1H3* and *ABCA1* expression levels were lower in adherent PBMCs from 22 consecutively seen SLE patients vs. 24 healthy controls (Figure 6A). As in pristane-induced lupus, *NR1H3* and *ABCA1* expression correlated in humans (Figure 6A). GW3965 treatment induced *ABCA1* and *NR1H3* expression in adherent PBMCs from healthy controls (Figure 6B). As in mice, *HIF1A* and *PFKL* expression levels were higher in adherent PBMCs from SLE patients vs. healthy controls (Figures 6C,D).

Systemic lupus erythematosus is associated with overproduction of IFNα/β (27). In the 22 consecutive SLE patients, CD64 fluorescence intensity on CD14^+^ cells, a marker of IFNα /β stimulation (28), was inversely associated with *ABCA1* expression (Q-PCR) (Figure 6E). CD64 surface staining also correlated inversely with ABCA1 intracellular staining intensity (flow cytometry) (Figure 6F). SLE patients with a SLEDAI ≥ 3 had low ABCA1 and high CD64 staining, whereas healthy controls exhibited the opposite pattern (Figure 6G).

**Figure 7:**
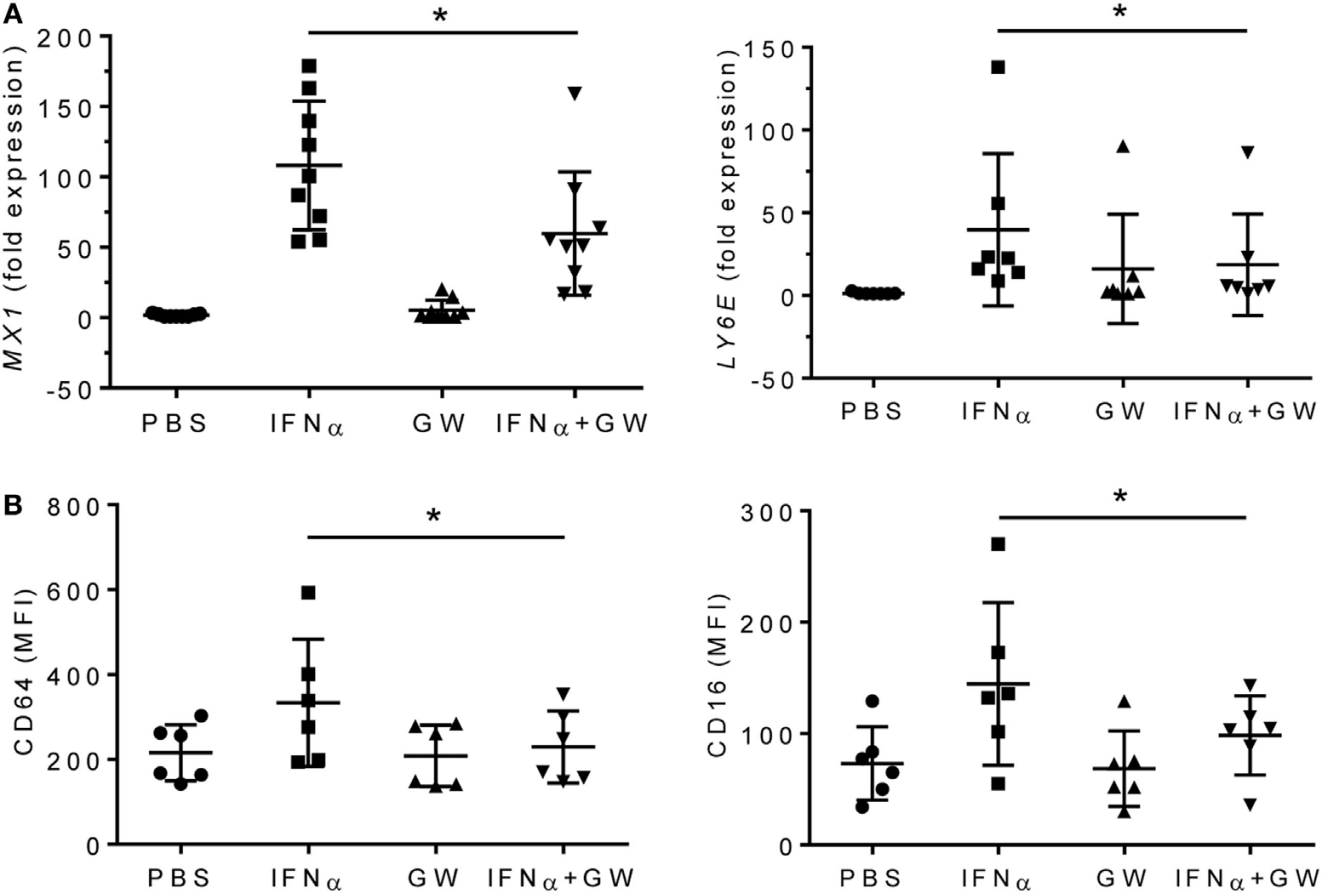
LXR agonist attenuates the type I interferon signature. Adherent PMBCs from healthy donors were incubated for 24 h with IFNα (1,000 U/ml), GW3965 (GW, 1 µM), or both. **(A)**, mRNA levels of *MX1* and *LY6E* were measured by Q-PCR. **(B)** CD64 and CD16 staining (mean fluorescence intensity, MFI) was determined by flow cytometry. *P < 0.05 vs. control (unpaired Student’s *t*-test).

To further examine the effects of LXRα activation on proinflammatory cytokines, we treated adherent PBMCs from healthy donors with IFNα or IFNα + GW3965 (Figure 7). GW3965 reduced expression of the IFN-I inducible genes *MX1* and *LY6E* (Figure 7A) and reduced fluorescence intensity of the IFN-I inducible surface markers CD64 and CD16 on CD14^+^ peripheral blood monocytes (Figure 7B), suggesting that LXR activation may downregulate the expression of interferon-regulated genes (interferon signature).

## Discussion

CD138^+^ Mϕ, which are highly phagocytic for apoptotic cells and promote the resolution of inflammation, are deficient in mice with pristane-induced lupus (6). This deficiency impairs the clearance of dead cells, a defect also seen in monocyte-derived Mϕ from SLE patients (29). We explored the possibility of treating lupus by enhancing the generation of these phagocytic CD138^+^ Mϕ. Consistent with their M2-like phenotype (6), CD138^+^ Mϕ from MO-treated mice had a metabolic profile consistent with alternatively activated Mϕ and expressed high levels LXRα, a transcription factor implicated in generating M2 Mϕ (13). In contrast, CD138^+^ Mϕ from pristane-treated mice were M1-like, expressing low levels of LXRα and high levels of HIF1α, a transcription factor that promotes glycolytic metabolism and the generation of M1 Mϕ (17, 22). Treatment of mice with pristane-induced lupus using an LXR agonist enhanced the expression of M2 Mϕ markers and prevented DAH, a severe inflammatory lung disease associated with pulmonary vasculitis that occurs in 3% of SLE patients (4, 30). Like PECs from pristane-treated mice, peripheral blood monocytes from SLE patients exhibited high HIF1α and low LXRα activity and LXR agonist treatment attenuated the interferon signature in these cells. The data suggest that abnormal Mϕ polarization contributes to the pathogenesis of SLE and that correcting the imbalance between M1- and M2-like Mϕ polarization may be a useful therapeutic strategy.

### M1–M2 Mϕ Imbalance in Pristane-Induced Lupus

We reported recently that a novel subset of CD138^+^ Mϕ with an M2 phenotype is highly phagocytic for apoptotic cells and promotes the resolution of inflammation. This subset is deficient in pristane-treated mice in comparison with MO-treated controls (6). In contrast, the M1-like Ly6C^hi^ Mϕ subset expands in pristane-treated mice. M1 Mϕ rely on glycolysis (high ECAR) whereas M2 Mϕ rely on fatty acid oxidation (high OCR) (15, 16). Mϕ from MO-treated mice had higher OCR, whereas ECAR was higher in pristane-treated mice (Figure 1), consistent with expansion of the M1 subset in pristane-induced lupus. Unexpectedly, CD138^+^ Mϕ from MO-treated mice had a higher OCR and expressed higher levels of M2 Mϕ markers [CD273, *Chil3* (Ym1), and IL-10] than those from pristane-treated mice, which preferentially expressed the M1 markers CD274, CD86, and TNFα (Figure 3). Thus, either the phenotype of CD138^+^ Mϕ subset exhibits some plasticity or there is more than one subset of CD138^+^ Mϕ. Our recent studies suggest the presence of an additional subset of proinflammatory CD138^+^ monocyte/Mϕ in pristane-treated B6 mice (S Han, unpublished data). Since HIF1α and LXRα regulate the gene expression programs of M1 and M2 Mϕ, respectively, we examined the activity of these transcription factors in pristane- vs. MO-treated mice.

### High HIF1α Activity in Lupus

Hypoxia-inducible factor 1-α and HIF1α-regulated genes were expressed at higher levels in both murine and human lupus (Figures 4 and 6). HIF1α is a hypoxia-induced regulator of glycolytic enzymes (e.g., HK2, PFKL, and G6PD) (17), and an inducer of M1 activation and the production of TNFα and other proinflammatory cytokines (18, 31). Heterodimers of HIF1α with the constitutively expressed aryl hydrocarbon receptor nuclear translocator bind and transactivate target genes containing hypoxia response elements (17). The transcriptional program induced by HIF1α is important for Mϕ and neutrophil function in infected (hypoxic) tissues (32). HIF targets include genes involved in aerobic glycolysis as well as inflammation (17, 33). The M1 marker CD274 (PD-L1) is HIF1α regulated and was expressed at higher levels in Mϕ from pristane- vs. MO-treated mice (Figure 3A).

### Impaired LXRα Activity in Lupus

In contrast to HIF1α, LXRα promotes M2 Mϕ development (13, 34). *Hif1a* mRNA expression correlated positively with *Tnfa* (Figure 1A) and inversely with the LXR-regulated gene *Abca1* (Figure 4B). Transcription factors of the LXR family form heterodimers with the retinoid X receptor, are activated by oxysterols (e.g., 25-hydroxycholesterol) (12), and regulate the transport of cholesterol transport to the liver and its biliary excretion (26, 35). Following uptake of apoptotic cells, oxysterols from the cell membranes activate the LXR pathway, upregulating the apoptotic cell receptor *MerTK* (14) and genes involved in cholesterol efflux (e.g., *ABCA1*). LXR activation downregulates innate immunity and inflammation by suppressing TLR signaling in Mϕ (12, 36). This may be one reason that phagocytosis of apoptotic cells is usually anti-inflammatory. Mice doubly deficient in LXRα and LXRβ exhibit proinflammatory signaling in response to apoptotic cells and develop lupus-like disease (14).

LXR activation is critical for M2 Mϕ polarization, expression of M2 signature genes, and downregulation of inflammation in activated Mϕ (34). In both pristane-induced lupus and SLE patients, expression of the LXR-regulated gene ABCA1 was impaired at both the RNA and protein level (Figures 2A and 6A). Lupus and control Mϕ did not exhibit substantially different *Nr1h3* gene expression, suggesting that the low Abca1 levels in lupus mice reflect impaired activation of LXR protein rather than low *Nr3h1* mRNA levels. However, our studies did not address the issue of whether the observed differences in Mϕ function specifically reflect the expression level of ABCA1 gene/protein or if the expression of other LXR-regulated genes plays a role. In mice, low LXRα was associated with high levels of TNFα and IFN-I regulated genes and low IL-10, especially in CD138^+^ Mϕ. In human monocytes, LXR agonists inhibited the induction of *MX1* and other type I IFN-stimulated genes by IFNα (Figure 7). Inhibition of *Hif1a* and *Pfkl* gene expression by LXR agonists (Figure 4C) further suggests that LXR may cross-regulate the HIF pathway, providing a potential mechanism for switching from M1 to M2 polarization.

### LXR Agonist Treatment Prevents DAH in Lupus

Our data suggested that HIF1α inhibitors or LXR agonists might benefit lupus patients by promoting M2 Mϕ polarization. Selective HIF1α inhibitors are not readily available, although there is interest in targeting the HIF1α activation pathway for cancer therapy (33, 37). Synthetic LXR agonists protect mice from atherosclerosis, myocardial ischemia-perfusion injury, and other conditions (26, 38). Unfortunately, their clinical use is complicated by hepatic steatosis, degradation of hepatic LDL receptors via the LXR-IDOL (inducible degrader of the LDL receptor) pathway, and/or unexplained neurological side effects (26, 38). However, the development of safer LXR agonists for clinical use is ongoing.

We gave pristane-treated mice the LXR agonist T0901317 to see if it could prevent DAH, an often fatal complication of SLE (2, 3). Daily LXR agonist treatment protected mice from DAH and promoted M2 repolarization of CD138^+^ Mϕ (Figure 5), suggesting that M1 Mϕ play a role in SLE-associated DAH. As DAH is similar in pristane-induced and human lupus (3), LXR agonists also might be useful in patients with DAH. We speculate that LXR agonists also might have a role in treating other Mϕ-mediated clinical manifestations of lupus. In lupus nephritis patients, glomerular and tubular Mϕ are among the best early correlates of proteinuria, declining creatinine, and poor renal outcome (39, 40). Mϕ also promote lupus nephritis in NZB/W mice (41, 42). Thus, lupus nephritis is a potential target for future testing of LXR-agonist therapy.

Low LXR expression also may be involved in accelerated atherosclerosis in SLE (43). Non-resolving inflammation in the vessel wall mediated by infiltrating Mϕ plays a central role in atherosclerosis and LXRs reciprocally regulate inflammation and lipid metabolism (34, 44). Similar to pristane-induced lupus (6), chronic inflammation in atherosclerotic plaques is associated with decreased non-inflammatory clearance of apoptotic cells by Mϕ (45). Thus, the LXR pathway may have far-reaching effects on the pathogenesis of organ damage in SLE.

Impaired Mϕ-mediated uptake of apoptotic cells is strongly associated with both human and murine lupus (6, 21, 29, 46). LXR signaling upregulates the clearance of apoptotic cells and its absence promotes autoimmunity (14). The present study provides the first evidence that LXR activity is abnormally low in monocytes/Mϕ from SLE patients whereas activity of HIF1α, a transcription factor that promotes inflammation and M1 polarization, is increased. The data support the clinical relevance of defective M1-M2 polarization, impaired apoptotic cell clearance, and non-resolving inflammation seen in pristane-induced lupus (6) and indicate that LXR agonist therapy aimed at repolarizing Mϕ can prevent disease, suggesting that a similar response may be achievable in SLE patients. LXR agonists modulated type I interferon production (Figure 7) and there is evidence for interplay between LXR signaling and Type I/Type II interferon production (47–49). However, LXR agonists are likely to have additional, interferon-independent, effects in lupus, since Type I interferon does not play a major role in the pathogenesis of DAH (3). It will be of interest to elucidate how signaling pathways downstream of LXR modulate the inflammatory response in lupus patients. Finally, the results identify imbalanced HIF1α and LXRα activity as a potential biomarker for assessing chronic inflammation in SLE patients and the response to anti-inflammatory therapy.

## Author Contributions

SH: Acquired the data and assisted in the analysis and interpretation and preparation of the manuscript. HZ: Acquired the data and assisted in the analysis and interpretation. SS: Assisted with data acquisition and analysis. JW: Assisted with data acquisition and analysis. CX: Assisted with data acquisition and analysis. HL: Assisted with data acquisition and analysis. LY: Assisted with data interpretation and preparation of the manuscript. WR: Responsible for the overall design of the study, analysis and interpretation of the data, and manuscript preparation.

## Conflict of Interest Statement

The authors declare that the research was conducted in the absence of any commercial or financial relationships that could be construed as a potential conflict of interest.
